# Teachers’ Perceptions of the Impact of the COVID-19 Pandemic and Their Implementation of an Evidence-based HIV Prevention Program in the Bahamas

**DOI:** 10.1007/s10461-024-04345-8

**Published:** 2024-04-20

**Authors:** Elizabeth Schieber, Lesley Cottrell, Lynette Deveaux, Xiaoming Li, Marcellus Taylor, Richard Adderley, Sharon Marshall, Nikkiah Forbes, Bo Wang

**Affiliations:** 1 Department of Population and Quantitative Health Sciences, UMass Chan Medical School, 368 Plantation Street, Worcester, MA 01605, USA; 2 Department of Pediatrics, West Virginia University, 959 Hartman Run Road. Morgantown, WVMorgantown 26506, USA; 3 Office of HIV/AIDS, Ministry of Health, Shirley Street, Nassau, Bahamas; 4 Department of Health Promotion, Education, and Behavior, University of South Carolina Arnold School of Public Health, 915 Greene Street, Columbia, SC 29208, USA; 5 Government and Public Policy Institute, University of The Bahamas, Oakes Field Campus University Drive, Nassau, Bahamas; 6 Department of Pediatrics, Wayne State University School of Medicine, 400 Mack Avenue, Detroit, MI 48201, USA

**Keywords:** Implementation Fidelity, Implementation Supports, HIV Prevention, School-based evidence-based Intervention, COVID-19

## Abstract

Information on how school-based programs is implemented and sustained during crises is limited. In this study, we assessed the impact of the COVID-19 pandemic on the implementation of a HIV prevention intervention in The Bahamas. Data were collected from 139 Grade 6 teachers in 2021–2022. Teachers attended virtual training and received implementation monitoring from coordinators. On average, teachers taught 26.4 (SD = 9.2) of the 35 core activities, and 7.4 (SD = 2.4) out of 9 sessions. More than half (58.3%) of teachers completed 28 or more core activities; 69.1% covered eight or all nine sessions, which is equivalent to 80% of the HIV intervention curriculum. Almost half of the teachers (43%) reported that the pandemic negatively impacted their ability to teach the program; 72% of teachers maintained that the program remained “very important” during times of crisis. Greater self-efficacy and supports increased implementation fidelity.

The COVID-19 pandemic disrupted the education sector worldwide, affecting 94% of the world’s students [1]. Since the pandemic’s onset, educational stakeholders have shared the need to acclimate to the changing educational landscape. School closures and adjustments to virtual and hybrid instruction led to significant loss of educational time [2]. Within that reduced instructional time, educators had to reexamine curricular priorities [3]. Sexual education teachers noted that sexual health and adolescent risk reduction curricula remained important and should continue to be covered despite barriers related to the COVID-19 pandemic, while recognizing that, in practice, sexual health education might not be considered a top priority during crises [4]. This prioritization against sexual health was also seen within the community as pandemic-related lockdowns interrupted public health programs such as child nutrition, mental health programming, sexual and reproductive health clinics, and HIV prevention and treatment programming [1–3].

## Teachers’ Roles During the Pandemic

Globally, teachers faced significant barriers when adjusting to the educational environment during the COVID-19 pandemic. Their roles in their schools and classrooms changed immensely as they adapted curricula to virtual and hybrid instruction and modified pedagogy to fit the needs of their students [5, 6]. Teachers with greater “digital literacy” (i.e., access and ability to navigate and use technology) were more likely to succeed with these adaptations [5, 7, 8]. Explicit training, peer mentorship, and practice using technology improved teachers’ digital literacy and adaptability during the pandemic [7, 9, 10]. However, the stress of adapting coursework combined with other pandemic-related stressors had large effects on teachers’ mental health and burnout [11, 12], necessitating supports to ease teachers’ burdens so they could best serve their students. Given the perceived importance of sexual health programming to prevent pregnancies and sexually transmitted infections [10, 11], teachers were also challenged with how to appropriately introduce and sustain program content virtually during this time.

## The Bahamas-Specific Barriers in Adapting to COVID-19 for Delivery of a School-based Prevention Program

In The Bahamas, over 102 school days were lost between 2019 and 2022 that had not been originally planned and were due to the pandemic [2]. Virtual school days were one hour shorter than regular school days [2]. An estimated 47% of students were unable to access online platforms at the beginning of the pandemic [13] and student attendance was abnormally low until December 2022 [14]. Further, the Virtual Learning Portal (a shared access cloud site that included space for curriculum materials, handouts, and other activities that each teacher wanted to store or share) only acted as a storage space for teachers, leaving them to pilot other options to host classes virtually (e.g., Zoom, Microsoft Teams) instead of creating a learning management system that would allow distribution of class materials and host virtual class meetings [2]. The Bahamas Ministry of Education (MOE) and teachers worked continuously and tirelessly to overcome these barriers and resume school activities, including a nationwide implementation of an adolescent risk-reduction program, *Focus on Youth in the Caribbean* (FOYC) and *Caribbean Informed Parents and Children Together* (CImPACT).

## FOYC + CImPACT

The FOYC + CImPACT program was designed for adolescents and their parents to discuss safe sex skills (e.g., condom use) and to avoid, or reduce, risky behaviors such as substance use, delinquency, and sexual risk behaviors [23–26]. Having been established as an evidence-based program within The Bahamas, we set out to test and validate implementation strategies that would possible offer flexibility for teachers [15]. These strategies incorporated biweekly monitoring and feedback (BMF) and site-based assistance and mentorship (SAM) to support teachers’ implementation of the program. BMF requires school coordinators to monitor teachers’ progress, give them feedback, and report any issues to the FOYC research office. For SAM, high-performing teachers became trained mentors to assist their peers in delivering the program. Additionally, we created a series of short videos that modeled implementing the FOYC core activities in a virtual format.

The implementation support study took place during the COVID-19 pandemic. Thus, faced several additional barriers that placed an emphasis on teachers to build rapport and promote virtual participation in the program while students tried to engage from their homes, which may have lacked privacy to discuss sexual health and related issues [9]. Little was known about Bahamian teachers’ perceptions of their experiences during the COVID-19 pandemic. Using data from the ongoing national implementation of FOYC + CImPACT, we explored: (1) teachers’ perceptions of the implementation barriers and facilitators created by the COVID-19 pandemic, (2) teachers’ views of the implementation supports we had initially developed for the study when put into place during a pandemic, and (3) how these perceptions influenced teachers’ implementation fidelity.

## Methods

### Study Site and Participants

Our study sample included 139 Grade 6 teachers representing 58 public elementary schools in The Bahamas. New Providence, the most populated island, had 80 teachers in 24 schools, and the Family Islands (including Grand Bahama) had 59 teachers in 34 schools. The research protocol was approved by the UMass Chan Medical School Institutional Review Board and the Institutional Review Board of the Bahamian Princess Margaret Hospital, Public Hospitals Authority.

### FOYC + CImPACT Program

FOYC consists of 30 core activities, to be taught in eight sessions to Grade 6 students during Health and Family Life Education (HFLE) classes throughout the school year, with annual booster sessions in Grades 7 through 9. The single CImPACT parent session has five core activities and was incorporated into parent-teacher meetings. For the combined program, Grade 6 teachers are expected to cover 35 core activities in 9 sessions. Teachers received BMF and SAM to support their program implementation.

### Teacher Training

Teachers were invited to attend a training webinar for FOYC + CImPACT, replacing the annual in-person training workshops. In three, 2-hour sessions, live instructor-led activities provided new FOYC + CImPACT teachers with the relevant curricular information and resources required to implement the program. Footage showed six Grade 6 teachers, who had experience implementing FOYC + CImPACT with high levels of fidelity, modeling the sessions and core activities of the program with 18 Grade 6 students. Trainers also conducted interactive didactic sessions to address sensitive questions that might arise in the classroom and increase teachers’ comfort with the material. Further, teachers received the situational analysis of HIV/AIDS and teen pregnancy in The Bahamas, a summary of FOYC efficacy research, an electronic copy of the FOYC + CImPACT manual, and access to online resources through the FOYC website. The webinar was delivered on the MOE’s Professional Development platform, and teachers could access recordings of it for reference. Sixty-one (76%) of the New Providence Grade 6 HFLE teachers attended the webinar; 16 (20%) of the teachers who did not attend the webinar had previously attended an FOYC + CImPACT training, and three (4%) teachers did not attend any training.

### School Coordinators and Peer Mentors

Two Bahamian trainers with extensive experience with FOYC + CImPACT conducted 2- to 3-hour training sessions with school coordinators and mentors. A school coordinator was identified for each of the schools and trained to provide BMF. The coordinators monitored teachers’ progress in covering the FOYC + CImPACT sessions and core activities and reported any issues to the FOYC research office in New Providence. Twelve high-performing teachers were identified to be mentors and trained to provide SAM to at-risk or moderate-performing teachers. They helped identify teachers’ challenges, assisted teachers in preparing for sessions, and provided guidance on curriculum delivery.

## Measures

### Implementation Fidelity

After each FOYC + CImPACT session, teachers completed a Teacher Implementation Checklist with which they documented which core activities were covered and whether they were taught exactly as outlined in the manual or whether any activities were modified. Teachers’ implementation fidelity was defined as the number of the 35 core activities of the FOYC + CImPACT curriculum taught throughout the year. Their degree of implementation was defined as the number of the 9 sessions completed. Trained observers attended ~ 10% of teachers’ sessions and completed the checklist to assess agreement between teachers’ and observers’ reports; agreement was high (~ 90%).

### Teachers’ Characteristics, Training Experience, and Perceptions

Teachers completed pre- and post-implementation questionnaires to provide information known to influence implementation fidelity and to reflect on their perceptions of their ability to implement the program. Only 107 teachers completed the pre-implementation questionnaire; 104 teachers completed the post-implementation questionnaire. Teachers reported their level of formal education, years as a teacher, training attendance, their perceptions of the importance of HIV prevention for Grade 6 students (very important, somewhat important, or not at all important), their comfort level teaching the FOYC + CImPACT curriculum (very comfortable, somewhat comfortable, or not comfortable at all), and any competing lessons or teaching priorities. Teacher perceptions of school support to teach the program was assessed using a 5-point Likert scale (1 = “totally disagree” to 5 = “totally agree”) with items such as “My principal expects FOYC to be taught to 6th graders.” [16, 17]. Teacher confidence was assessed across five areas “I feel like I can complete teaching the whole FOYC program.” based on a 5-point Likert scale (1 = “not at all confident” to 5 = “very confident”) [18]; and eight items assessing *attitudes toward sex education* in schools (e.g., “School is the ideal place to teach sex education as one of many different aspects of education.”) [19]. The internal consistency (Cron-bach’s α) of the scales is adequate (principal supportiveness α = 0.85; confidence α = 0.91; attitudes toward sex education α = 0.68). Further, teachers reflected on whether they were able to complete the entire FOYC + CImPACT program and what factors prevented completion of the program (e.g., “preparation time; other priorities”) through open text capture including the level of impact COVID-19 may have had based on a 5-point Likert scale (1 = “strong negative impact” to 5 = “strong positive impact”) and whether they found FOYC important to teach during a public health crisis such as the COVID-19 pandemic (“it is not important to teach FOYC during a crisis,” “somewhat important,” or “very important.”).

### School Coordinators’ and Mentors’ Performance

School coordinators’ and mentors’ performance of BMF and SAM were monitored by the national school coordinator, the New Providence coordinator, The Bahamas FOYC project manager, and the FOYC curriculum trainers. Using a 14-item questionnaire, evaluators ranked school coordinators’ and mentors’ performance annually as “Unsatisfactory,” “Satisfactory,” “Good,” or “Excellent.” School coordinators were assessed on their knowledge of teachers’ implementation activities, communication with the FOYC research office, and submission of required materials. Mentors were rated for their performance, and the number of mentoring sessions they held during the academic year was noted.

### Conceptual Model

**Fig. 1:**
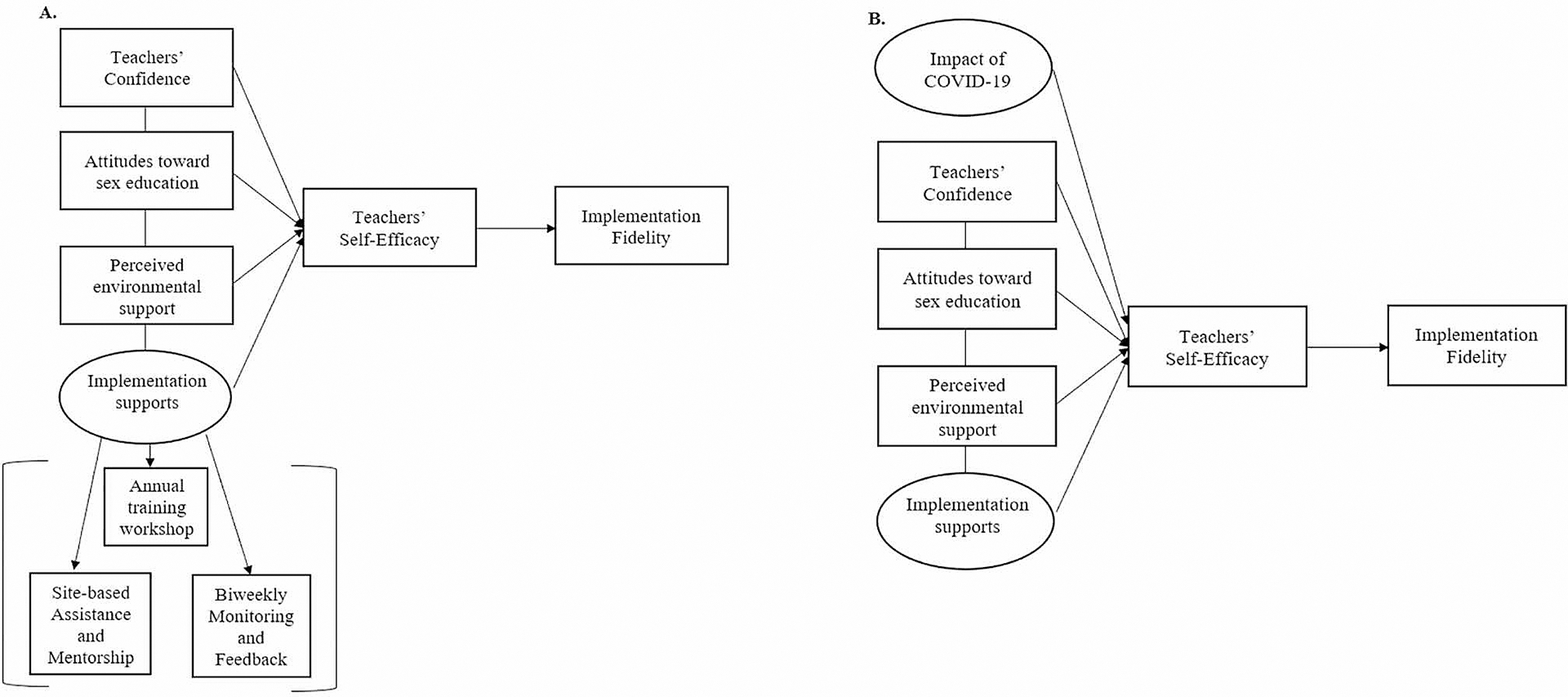
The conceptual diagram of the program components’ effects on teachers’ implementation fidelity before the COVID-19 pandemic (**A**) and including the impact of COVID-19 (**B**)

As noted earlier, we had been implementing additional teacher supports for implementing the program when the pandemic occurred (see Fig. 1a), which increased the importance of examining the potential effect those supports had for teachers as they shifted to remote learning for all their subjects. The additional supports and strategies were designed and implemented to strengthen teacher self-efficacy originally but may have had a different effect during the pandemic when competing challenges were noted (Fig. 1b).

## Analysis

### Quantitative Analyses

First, using SAS 9.4 [20], we examined (by ANOVA, t test, and chi-square test) the association between teachers’ perceptions of the impact of the COVID-19 pandemic on their ability to fulfill their teaching roles, perceived importance of teaching FOYC during a crisis, school coordinator’s support, online training, and performance of school coordinators and mentors with teachers’ implementation of FOYC + CImPACT (e.g., number of core activities and number of sessions taught). Second, Spearman correlation analysis was used to examine associations between implementation degree and factors influencing teachers’ implementation using SAS 9.4 [20]. Third, structural equation modeling (SEM) analysis was conducted to examine the relations among factors influencing teachers’ implementation (including teachers’ perceptions of the impact of the COVID-19 pandemic, confidence, comfort level, attitudes toward sex education in schools, perceived principal support, school coordinator performance, self-efficacy) using Mplus 8 [21]. Standardized regression coefficients for all paths were estimated using robust maximum likelihood (MLR) estimation. Goodness of model fit was evaluated using chi-square to degrees-of-freedom ratio (χ^2^/df), root mean square error of approximation (RMSEA), Bentler’s comparative fit index (CFI), and the Tucker Lewis Index (TLI) [22]. Acceptable model fit is determined by an RMSEA less than 0.08 and values of CFI and TLI greater than 0.90 [22].

### Enumeration of Themes from free text Responses

We conducted a thematic analysis [23] of the free-response questions on the post-implementation questionnaire to discern any common themes of what teachers perceived as barriers and facilitators for teaching FOYC. One study team member created the initial set of codes, using inductive coding to create the code book. Then a second team member used deductive coding. Any discrepancies were discussed between the coders and reconciled.

## Results

### Teachers’ Implementation of FOYC + CImPACT Program

On average, teachers taught 26.4 (SD = 9.2) of the 35 core activities, and 7.4 (SD = 2.4) out of 9 sessions. Out of 139 teachers, 81 (58.3%) teachers completed 28 or more core activities, and 96 (69.1%) covered eight or all nine sessions, which is equivalent to 80% of the HIV intervention curriculum. Twelve (8.6%) teachers covered fewer than 3 sessions; thirteen (9.4%) teachers taught fewer than 10 core activities.

### Bivariate Association of Teachers’ Perceptions of the Impact of the COVID-19 Pandemic with Implementation of FOYC + CImPACT

As shown in Table 1, teachers who perceived their school coordinators as very supportive of teaching FOYC taught more core activities than other teachers. Teachers who thought online training was not as effective as an in-person training workshop taught more sessions (8.8 vs. 7.8, *t* = 4.24, *p* < 0.001) and core activities (32.0 vs. 27.9, *t* = 3.73, *p* < 0.001). In addition, the island where a teacher worked was significantly associated with that teacher’s implementation. Teachers who worked on the capital island (New Providence) taught more sessions and core activities than teachers who worked in the Family Islands (sessions: 8.0 vs. 6.5, *t* = 3.44, *p* < 0.001; core activities: 29.3 vs. 22.4, *t* = 4.40, *p* < 0.001). Teachers’ perceptions of the impact of the COVID-19 pandemic on their ability to fulfill their teaching roles, perceived importance of teaching FOYC during a crisis, and school coordinator follow-up were not significantly associated with teachers’ implementation degree.

### Bivariate Association of School Coordinators’ and Mentors’ Support with Implementation of FOYC + CImPACT

As shown in Table 2, teachers who had a “very good” or “excellent” school coordinator taught more core activities (28.9 and 28.1 on average) than those who had a “satisfactory” coordinator (22.4 on average) or no coordinator (22.3 on average) (*F* = 4.43, *p* < 0.01). Teachers who worked in a school that had a trained FOYC mentor or who were mentored by teachers from the same school taught more core activities and sessions than teachers who did not have a mentor (core activities: 26.0 and 30.0 vs. 24.1; *F* = 5.52; *p* < 0.001. sessions: 7.4 and 8.2 vs. 6.9, *F* = 3.34, *p* < 0.05).

### Correlations among Factors Influencing Teachers’ Implementation of FOYC + CImPACT

The strength of associations between implementation and factors influencing teachers’ self-efficacy was examined using Spearman correlation coefficients (Table 3). Teachers’ perceptions of the impact of the COVID-19 pandemic on their ability to fulfill their teaching roles (including FOYC, CImPACT, and other subjects) were negatively related to their own self-efficacy (*r* = −0.25 to −0.39, *p* < 0.001). Teachers’ comfort level with the curriculum, confidence in teaching core activities, attitudes toward sex education in schools, perceived principal support, and school coordinator support/performance were significantly related to greater self-efficacy (*r* = 0.43 to 0.62, *p* < 0.001). Teachers’ confidence, attitudes toward sex education, school coordinator support/performance, and self-efficacy were significantly related to the number of core activities taught (*r* = 0.21 to 0.30, *p* < 0.05). Teachers’ perceptions of the impact of the COVID-19 pandemic were negatively related to teachers’ comfort level (*r* = −0.22 to −0.32, *p* < 0.05) and confidence in teaching core activities (*r* = −0.24 to −0.31, *p* < 0.05). Teachers’ comfort level was significantly related to teachers’ confidence, positive attitudes toward sex education in schools, perceived principal support, and school coordinator support (*r* = 0.34 to 0.65, *p* < 0.001). Teachers’ confidence was also related to their perceptions of the role of sex education in school curricula, perceived principal support, and school coordinator support (*r* = 0.20 to 0.43, *p* < 0.05). In addition, school coordinator support was related to teachers’ attitudes toward sex education and perceived principal support (*r* = 0.37 to 0.38, *p* < 0.001).

### Relations among Factors Influencing Teachers’ Implementation, Self-efficacy, and Implementation of FOYC + CImPACT

Structural equation modeling demonstrated relations among the factors and their direct and indirect effects on implementation fidelity (i.e., number of core activities taught) (Fig. 1). The model involved one latent exogenous variable (impact of COVID-19), five manifest exogenous variables (teachers’ comfort level, teachers’ confidence, attitudes toward sex education, perceived principal support, and performance of school coordinator), and two manifest endogenous variables (self-efficacy, implementation degree). In modifying the initial model, we removed the paths from teachers’ comfort level, teachers’ confidence, and attitudes toward sex education, the impact of COVID-19 to implementation fidelity, and the path from teachers’ confidence to self-efficacy as they were nonsignificant.

In the revised model, teachers’ comfort level, attitudes toward sex education, perceived principal support, and performance of school coordinator predicted greater self-efficacy, which in turn predicted higher implementation. Teachers’ self-efficacy and performance of school coordinator had positive direct effects on implementation degree. Teachers’ comfort level was positively related to teachers’ confidence and perceived principal support. The impact of COVID was negatively related to teachers’ confidence and comfort level. The overall fit of the revised SEM model was excellent (CFI = 0.987, TLI = 0.975, RMSEA = 0.05, χ^2^/*df* = 1.35; *p* = 0.146; SRMR = 0.04). The analysis revealed R^2^ values of 0.55 for teachers’ self-efficacy and 0.16 for implementation fidelity.

The mediation effect analysis indicated that self-efficacy mediated the relation between the perceived impact of the COVID-19 pandemic, teacher’s level of comfort with the FOYC curriculum, perceived principal support, and teachers’ implementation degree (impact of COVID: *z* = 2.00, *p* = 0.045; comfort: *z* = 2.05, *p* = 0.04; principal support: *z* = 2.01, *p* = 0.044).

### Synthesis of Teachers’ Perceptions

Themes, subthemes, and representative quotes for the free-response questions are summarized in Table 4.

#### “What did you Enjoy/like Most About Teaching FOYC?”

Three themes emerged from the teachers’ responses to this question: [1] Students’ engagement [2], Students’ learning outcomes, and [3] FOYC + CImPACT activities and program structure. A large majority of teachers (71.1%) commented on their students’ engagement during FOYC sessions, and that the interactive nature of the activities evoked class participation and deep discussions. Some teachers (34.2%) also expressed appreciation for the learning outcomes for the students; 25% of teachers noted that the life skills taught during FOYC + CImPACT are beneficial for adolescents’ development, and 9.2% explicitly noted the importance of sex education. Further, 18.4% of teachers responded that the program’s structure and materials made teaching the sessions easier.

#### “Why Would Some Grade 6 Teachers Not Want to Teach the FOYC Curriculum?”

Teachers’ responses to this question also revealed three themes: [1] sensitive content [2], curricular priorities, and [3] perceptions that it should not be the teacher’s duty to cover these topics. Most teachers (81.6%) hypothesized that some teachers may be uncomfortable with content covered during FOYC + CImPACT sessions, which could result in their not wanting to teach some or all the curricula. Several teachers (18.4%) noted that teachers may prioritize other subjects and preparation for standardized exams over FOYC + CImPACT. Further, 9.2% expressed that some teachers may perceive the content would be better covered by parents, guidance counselors, or nurses instead of Grade 6 teachers. Of note, one teacher explicitly stated that she did not enjoy teaching FOYC + CImPACT.

#### “What are the Challenges that Some Teachers may have when Engaging Grade 6 Students in Frank Discussion on Sensitive Topics?”

Three themes also emerged from teachers’ responses to this question: [1] a lack of comfort for students and/or teachers [2], concerns about the content, and [3] lack of challenges when engaging students in discussion. Most teachers (69.7%) expressed that either teachers or students may be uncomfortable with the subject matter. Only 13.2% of teachers expressed that other teachers may have issues hosting these discussions, but 56.6% noted that some students may be too shy, immature, or generally uncomfortable with sensitive discussions. Several teachers (32.9%) expressed concern about the content: its appropriateness for Grade 6 students (22.4%), issues parents have raised about the content (5.3%), and teachers’ ability to cover all the content and still meet students’ needs (5.3%). A few teachers (5.3%) did not perceive that teachers should have any challenges with these discussions.

#### “How did the Virtual Environment Facilitate Teaching FOYC?”

Responses to this question fell into three themes: [1] accessibility [2], access to online resources, and [3] absence of facilitation in the virtual environment. Some teachers (14.5%) noted that the virtual environment provided accessibility to teach the program during the COVID-19 pandemic, with 2.6% of teachers noting it was a way to access their students, 4.0% noting it allowed them access to parents, and 6.6% of teachers expressing that some students were more comfortable at home. Many teachers (47.4%) expressed that the online resources created for FOYC + CImPACT enabled them to teach the sessions virtually. However, 38.2% of teachers expressed that the virtual environment was not conducive to teaching FOYC + CImPACT, and 9.2% explicitly stated they preferred teaching the program face-to-face.

#### “What Challenges did you Experience when Teaching FOYC in the Virtual Environment?”

Six themes arose from the responses to this question [1], student interaction [2], technical difficulties [3], some activities were not suitable for virtual instruction [4], students’ home environments [5], absence of challenges, and [6] did not teach FOYC virtually and waited to return to school. Most teachers (59.2%) expressed that student interaction was lower because of poor attendance (30.3%), reduced engagement (23.7%), or difficulties establishing rapport with students (5.3%). Many teachers (31.6%) noted that technical difficulties presented challenges because of issues with Wi-Fi connections (26.3%), reduced instruction time during virtual school days (3.9%), and their own comfort navigating the technology (1.3%). Further, 10.5% of teachers responded that some of the FOYC + CImPACT activities were not as well suited for virtual instruction as for in-person. Many teachers (30.3%) noted that students’ home environments were not always conducive to teaching FOYC because the presence of others in the home might have affected students’ comfort in engaging in the activities and discussions. Although 9.2% of teachers reported that they had no challenges teaching the program virtually, 2.6% chose not to teach FOYC virtually and instead waited for schools to return to in-person.

## Discussion

The COVID-19 pandemic posed challenges for the implementation of FOYC + CImPACT in The Bahamas. We examined teachers’ perceptions of the pandemic’s impacts on their ability to teach the program and found that teachers who perceived negative impacts from the pandemic on their ability to teach the program had lower comfort levels, confidence, and self-efficacy. However, higher self-efficacy predicted greater implementation and mediated the relation between teachers’ perceived impacts of COVID-19 and their degree of implementation even during the pandemic. Teachers’ comfort levels, attitudes toward sex education, and perceived principal support, and school coordinators’ performances providing BMF increased their self-efficacy and, in turn, implementation fidelity. Our implementation supports, especially BMF and annual training to increase teachers’ comfort with and attitudes about the curriculum, and administration buy-in from principals may have facilitated teachers’ implementation of FOYC + CImPACT nationwide. On average, teachers taught ~ 75% of the curriculum despite barriers during the pandemic, providing their students with opportunities to learn critical skills to reduce risk behaviors. Teachers in New Providence on average taught more sessions and covered more core activities than those in the Family Islands, and the New Providence teachers were more likely to have access to school coordinators, peer mentors, and other structural supports (e.g., strong broadband) than those in the Family Islands. Based on these findings, we would propose to continue to include local supports from the school environment but also mentorship and modeling that could be provided virtually (as was done in this study) by coordinators.

Most of the Grade 6 teachers in The Bahamas (72%) agreed that continuing to teach FOYC + CImPACT during times of crisis such as the COVID-19 pandemic remained “very important,” which is supported by experts’ opinions [4, 24, 25]. FOYC + CImPACT also teaches students decision making and assertive communication skills to prevent not only sexual risk behaviors, but also substance use and delinquency. These skills remain essential and are perhaps of even greater importance during times of crisis. Teachers in this study reported the continued importance and prioritization of these types of programs during a pandemic. Those who perceived it as a priority also had high implementation fidelity. Additionally, coordinator supports such as the BMF in this study continued to review the importance (and ease) of implementing the program during a different and potentially chaotic time for the teachers.

Although 71% of our teachers stated that engaging the students during FOYC sessions was what they enjoyed most about the program, 59% noted that interacting with students was especially challenging during virtual instruction. Students across the globe experienced mental health issues during the pandemic because of social isolation [26]. They were less likely to engage in virtual instruction [27]. An analysis found that German students’ engagement in virtual instruction decreased, and dropout rates increased [28]. Further, school closures exacerbated socioeconomic inequalities [29]. For example, students with weaker internet at home were less likely to be able to interact during virtual instruction [8]. While teachers were exploring how to best engage their students online [4, 30], students also had to learn digital literacy and potentially overcome interpersonal barriers at home [27]. Future studies would advance this work by identifying strategies to engage youth in virtual programs. These strategies may be like those in the BMF and other successful supports with teachers in this study. Explicit training for teachers and students to navigate virtual instruction may help mitigate additional technical issues in the future [9, 10], but structural inequalities such as internet access also need to be addressed, especially in small-island developing states like The Bahamas [31].

### Limitations

Despite highlighting teachers’ perspectives about the implementation of FOYC + CImPACT during the COVID-19 pandemic, this study has several limitations. The phrasing of some of the open-ended questions may have biased some teachers’ responses. Namely, “What are the challenges that some teachers may have when engaging Grade 6 students in frank discussions on sensitive topics?” and “Why would some Grade 6 teachers not want to teach the FOYC curriculum?” solicited conjecture of *others’* potential experiences instead of the respondents’ personal accounts. Further, teachers’ self-reported data are subject to response bias and reactivity. Another limitation: we did not collect students’ outcome data, though the relation between student outcomes and implementation fidelity has been established for FOYC + CImPACT [32, 33]. The current analyses aimed to measure teachers’ perspectives and the effects of those perspectives on their implementation of FOYC + CImPACT in a nationwide implementation of the program.

## Conclusions

Experts have stressed the importance of increasing teachers’ self-efficacy and flexibility when adjusting to new educational challenges [10]. Performance of school coordinators providing BMF both increased teachers’ self-efficacy and directly influenced their implementation. Our data confirm that self-efficacy was a large predictor of teachers’ degree of implementation. This result highlights the importance of providing implementation supports that can enhance self-efficacy to improve teachers’ performance. Planning for robust implementation strategies that can address changing educational landscapes can increase the resilience of public health programs such as FOYC + CImPACT in the face of external challenges such as the COVID-19 pandemic.

## Figures and Tables

**Fig. 2:**
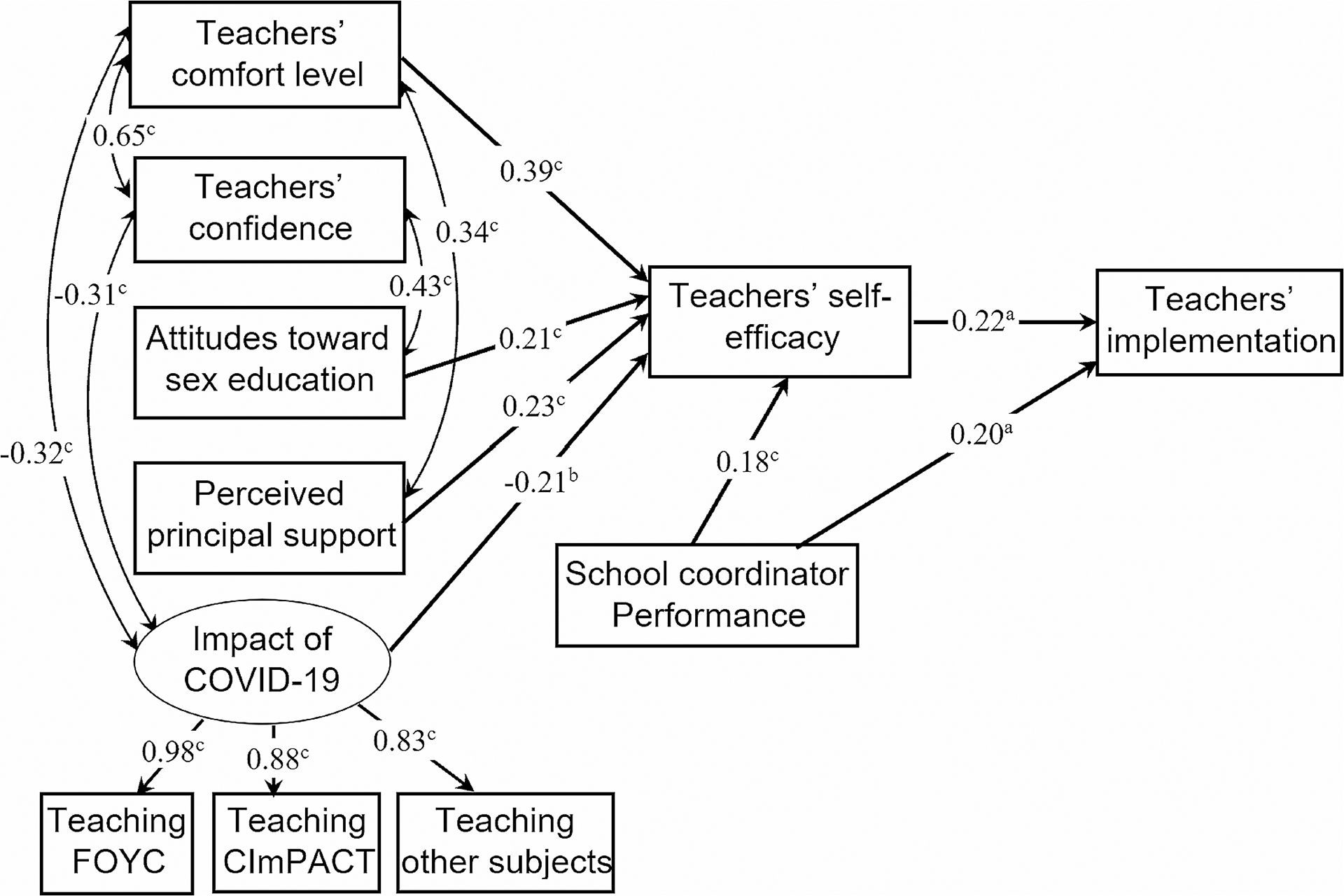
Revised structural model showing relationships among factors influencing teachers’ implementation. Standardized path coefficients are shown Note: a *p* < 0.05; b *p* < 0.01; c *p* < 0.001

**Table 1:** Association between perceptions of the impact of the COVID-19 pandemic, online training, and teacher implementation dose and fidelity among Grade 6 teachers (*N* = 107)

Variables	No. of teachers	Number of sessions taught (0–9) M (SD)	Number of core activities taught exactly as outlined (0–35) M (SD)	Number of core activities completed (0–35) M (SD)
The COVID pandemic impacted your ability to fulfill your teaching roles for FOYC				
Negative impact	46	8.3(1.5)	29.6(6.1)	17.8(9.3)
Not much of an impact	34	7.9(1.3)	28.9(5.6)	20.6(9.7)
Somewhat of a positive impact	27	7.9(2.1)	28.0(8.3)	19.7(10.4)
F test		1.03	0.49	0.90
The COVID pandemic impacted your ability to fulfill your teaching roles for CImPACT				
Negative impact	36	8.2(1.7)	29.2(6.7)	17.5(9.6)
Not much of an impact	49	7.9(1.4)	28.8(5.9)	19.7(9.7)
Somewhat of a positive impact	22	8.1(2.0)	29.1(7.9)	20.8(9.7)
F test		0.35	0.06	0.89
The COVID pandemic impacted your ability to fulfill your teaching roles for other school subjects				
Negative impact	50	8.4(1.5)	29.6(6.2)	18.0(9.7)
Not much of an impact	33	7.9(1.4)	29.0(5.5)	19.6(9.3)
Somewhat of a positive impact	24	7.7(2.2)	27.7(8.6)	21.1(10.3)
F test		1.40	0.66	0.80
Importance to teach FOYC during a crisis such as the COVID-19 pandemic or national disaster				
Not important	8	8.6(0.7)	30.0(6.2)	17.1(9.2)
Somewhat important	22	8.2(1.2)	28.6(5.0)	19.9(6.9)
Very important	77	8.0(1.8)	29.0(7.0)	19.2(10.4)
F test		0.62	0.14	0.23
My school coordinator is supportive of teaching FOYC				
Either agree or disagree	11	8.5(0.7)	28.9(4.6)	18.9(7.1)
Agree	29	7.5(2.1)	26.5(7.8)	15.9(10.6)
Strongly agree	67	8.2(1.4)	30.0(6.0)	20.7(9.4)
F test		2.31	3.00*	2.45
My school coordinator follows up on my progress in teaching FOYC (keep or not)				
Either agree or disagree	14	8.5(0.7)	30.0(3.4)	21.0(6.8)
Agree	33	7.7(2.0)	27.2(7.9)	16.9(10.4)
Strongly agree	60	8.2(1.5)	29.8(6.1)	20.1(9.7)
F test		1.52	1.86	1.37
Online training is not as effective as an in-person training workshop				
Yes	27	8.8(0.6)	32.0(3.8)	20.7(9.4)
No	80	7.8(1.8)	27.9(7.0)	18.7(9.8)
t test		4.24***	3.73***	0.94
Island				
New Providence	80	8.0(1.6)	29.3(6.5)	19.8(10.3)
Family Islands	59	6.5(3.0)	22.4(10.8)	14.0(9.9)
t test		3.44^c^	4.40^c^	3.31^b^

Note:

* *p* < 0.05;

** *p* < 0.01;

*** *p* < 0.001.

**Table 2:** Association between performance of school coordinators and mentor and teacher implementation of FOYC + CImPACT among Grade 6 teachers (*N* = 134)

Variables	No. of teachers	Number of sessions taught (0–9) M (SD)	Number of core activities taught exactly as outlined (0–35) M (SD)	Number of core activities completed (0–35) M (SD)
School coordinator’s performance				
No School coordinator [1]	10	6.3(2.9)	22.3(10.8)	15.4(10.6)
Satisfactory [2]	24	6.1(2.7)	22.4(10.4)	18.5(11.5)
Very Good [3]	45	8.2(1.7)	28.9(6.4)	18.4(9.4)
Excellent [4]	55	7.9(2.1)	28.1(8.7)	17.3(10.6)
F test		6.32***	4.43**	0.31
Pair-wise comparisons		[1–4]	[2–4]	
Having a peer mentor				
No mentor [1]	37	6.9(3.0)	24.1(10.5)	15.4(10.3)
Mentored by teachers from the same school [2]	48	8.2(1.4)	30.0(6.1)	20.8(9.9)
Mentored by a trained FOYC mentor [3]	49	7.4(2.2)	26.0(9.0)	16.6(10.1)
F test		3.34*	5.52**	3.55*
Pair-wise comparisons		[1, 2]	[1, 2]	[1, 2]

Note:

* *p* < 0.05;

** *p* < 0.01;

*** *p* < 0.001.

**Table 3:** Bivariate correlations among factors influencing teachers’ continued implementations

Variables	1	2	3	4	5	6	7	8	9	10	11	12	
1. Number of sessions taught	1.00												
2. Number of core activities taught	0.87^c^	1.00											
3. Number of core activities taught exactly as outlined	0.47^c^	0.57^c^	1.00										
4. Attitudes toward sex education in schools	0.12	0.23^a^	0.11	1.00									
5. Perceived principal support	−0.04	0.14	0.03	0.15	1.00								
6. School coordinator’s support	0.18	0.27^c^	0.18	0.37^c^	0.38^c^	1.00							
7. Confidence	0.03	0.21^a^	0.24^a^	0.43^c^	0.20^a^	0.37^c^	1.00						
8. Comfort level	−0.11	0.09	0.11	0.37^c^	0.34^c^	0.35^c^	0.65^c^	1.00					
9. Self-efficacy	0.13	0.30^b^	0.14	0.43^c^	0.44^c^	0.44^c^	0.48^c^	0.62^c^	1.00				
10. COVID-19 pandemic impacted my ability to teach FOYC	0.13	0.03	−0.10	−0.10	−0.18	−0.17	−0.31^c^	−0.32^c^	−0.39^c^	1.00			
11. COVID-19 pandemic impacted my ability to teach CImPACT	0.04	−0.03	−0.11	−0.06	−0.14	−0.15	−0.24^a^	−0.22^a^	−0.37^c^	0.88^c^	1.00		
12. COVID-19 pandemic impacted my ability to teach other subjects	0.15	0.05	−0.12	−0.01	−0.12	−0.12	−0.24^a^	−0.22^a^	−0.25^b^	0.82^c^	0.74^c^	1.00	
13. School coordinators’ performance	0.33^c^	0.25^b^	−0.02	0.09	0.04	0.43^c^	0.11	0.02	0.21^a^	0.01	−0.02	0.10	1.00

Note:

a *p* < 0.05;

b *p* < 0.001;

c *p* < 0.001.

SD = Standard deviation. Score range: 1–5 for sex education. principal support, confidence, comfort level, and self-efficacy.

**Table 4:** Quotes from open-ended questions

Theme Subthemes	% of teachers (*N* = 104)	Representative quotes
**How did the virtual environment facilitate teaching FOYC?**		
Accessibility	14.5%	“The virtual environment facilitate FOYC by allowing students access to learning while at home.” “It made the sharing of videos & other visual aids fun for the students & easily accessible for me.” “It help [sic] a lot because many of their guardians were at home to add to the conversation.” “The students were more comfortable to interact with their teachers.”
Students more	6.6%	
comfortable	2.6%	
Can reach kids during	4%	
C19	1.3%	
Can meet with Parents		
More time for topics		
No Facilitation	38.2%	“Unfortunately the virtual environment didn’t facilitate the FOYC teaching much. It limited what was discussed and how the discussion came out.” “Not well; it was impersonal” “The session was fine virtually but could’ve been better had it been face to face” “I was okay, but I prefer face to face due to the sensitivity of the topics and the freedom to cover the topics with a better feeling of the kids.”
Prefer face-to-face	9.2%	
	
	
	
Access to online resources (for students and teachers)	47.4%	“It was easier to send links and show videos and play games while students were able to view and play from their own devices.”
**What challenges did you experience when teaching FOYC in the virtual environment?**		
Student interaction	59.2%	“Not having (physically) met my students, I struggled in the beginning with forming a connection through learning their personalities. It was difficult to gauge their level of understanding. Participation was also very low.” “It was difficult to gage whether or not the students were focused on the lesson. It was also difficult to do some of the activities, which once in person, the students really enjoyed,”
Poor attendance	30.3%	
General engagement	23.7%	
Building rapport	5.3%	
Technical difficulties	31.6%	“A challenge for me was a lot of the students were missing for some technical reason or the other.”
Wifi/tech issues Teacher	26.3%	
comfort	1.3%	
Not enough time	3.9%	
Activities difficult virtually	10.5%	“Some of the role playing and group activities were a challenge.” “Not being able to play the games and get questions of concerns from the students.”
Home environment/others in home	30.3%	“Having the frank conversations because parents were listening in so students didn’t want to open up.” “Some students had a great deal of distractions at home and some were uncomfortable talking about certain things at home in the presence of a parent or family member” “There were too many people in the background that felt that the information being discussed was inappropriate for grade 6 and the younger children in the background. Some parents told children to logout during FOYC”
Did not teach FOYC virtually/waited for in-person	2.6%	“I didn’t teach FOY in the virtual environment. We did it face to face after we returned in 2022”
No challenges	9.2%	“None, I was always enthused and extremely excited to teach the FOYC curriculum”
**What did you enjoy/like most about teaching FOYC?**		
Student Engagement	71.1%	“I enjoyed teaching all of the topics because my students were very engaged in the discussions and were curious about a lot, so I was glad to be able to clarify information for them.”
Student learning	34.2%	“The idea that I could help children make good decision is what inspires me about the program. I enjoyed the goal setting section the most.” “Educating students on sex education before their peers or social does it.” “Sharing all of what the program has to offer our students, I am a firm believer that a young life saved is a whole life saved. I enjoy teaching the program to our students.” “Building students self esteem and values, exposing them to do’s and don’ts of sex” “I enjoyed the activities provided in the Focus on Youth resources.”
outcomes	25%	
Life Skills	9.2%	“The idea that I could help children make good decision is what inspires me about the program. I enjoyed the goal setting section the most.” “Educating students on sex education before their peers or social does it.” “Sharing all of what the program has to offer our students, I am a firm believer that a young life saved is a whole life saved. I enjoy teaching the program to our students.” “Building students self esteem and values, exposing them to do’s and don’ts of sex” “I enjoyed the activities provided in the Focus on Youth resources.”
Sex education	
Program activities and structure	18.4%	“I enjoyed the activities provided in the Focus on Youth resources.”
**What are the challenges that some teachers may have when engaging Grade 6 students in frank discussions on sensitive topics?**		
Comfort	69.7%	“Some students tend to shy away from engaging in certain aspects of these topics. Others can overreact and become very loud or begin laughing and taking crucial time away from the sessions.”
Of students	56.6%	
Of teachers	13.2%	“Religious beliefs may impact how teachers engage with the students. Also, some teachers may be uncomfortable with these sensitive topics” “Content of topic being too intense. Let the guidance or a specialist teach it.” “The challenge I saw occurring this year was that of some parents not wanting other than them to teach certain aspects of the program to their child.” “Challenges faced by most teachers may have been time constraints. Frank discussions tend to go longer and deeper” “There really should not be any if they are trying to help the students.” “There aren’t any challenges as grade 6 students are always interested in discussions.”
Content	32.9%	“Content of topic being too intense. Let the guidance or a specialist teach it.”
Appropriateness of	22.4%	“The challenge I saw occurring this year was that of some parents not wanting other than them to teach certain aspects of the program to their child.” “Challenges faced by most teachers may have been time constraints. Frank discussions tend to go longer and deeper” “There really should not be any if they are trying to help the students.” “There aren’t any challenges as grade 6 students are always interested in discussions.”
content	5.3%	
Parental concerns	5.3%	“Challenges faced by most teachers may have been time constraints. Frank discussions tend to go longer and deeper”
Time to teach it all		
No challenges	5.3%	“There really should not be any if they are trying to help the students.” “There aren’t any challenges as grade 6 students are always interested in discussions.”
**Why would some Grade 6 teachers not want to teach the FOYC curriculum?**		
Sensitive content	81.6%	“Some grade 6 Teachers may not want to teach the FOY curriculum because of the sensitive nature of the of the content.” “I believe some persons do not feel the children should knowledgeable about sex at an early age.”
Curricular Priorities	18.4%	“Grade 6 has a lot of content and preparation for GLAT Exam for the students. GLAT Preparation are time consuming.” “They may not want to teach the curriculum because sometimes it takes time away from other core subjects.”
Not the teacher’s place to cover	9.2%	“Due to the depth of teaching children about sex, and many think its the parents job.”
